# Developing a Suicide Risk Prediction Algorithm Using Electronic Health Record Data in Mental Health Care: Real-World Case Study

**DOI:** 10.2196/74240

**Published:** 2026-01-14

**Authors:** Linda Hummel, Karin C A G Lorenz-Artz, Joyce J P A Bierbooms, Inge M B Bongers

**Affiliations:** 1 Tranzo Scientific Center for Care and Wellbeing, Tilburg School of Social and Behavioral Sciences, Tilburg University, Prof. Cobbenhagenlaan 125, Reitse Poort, Room RP 204, Tilburg, 5037 DB, The Netherlands, 31683662495; 2 GGzE Mental Health Care Institute, Eindhoven, The Netherlands

**Keywords:** implementation science, artificial intelligence, prediction algorithms, electronic health records, mental mealth services, suicide prevention

## Abstract

**Background:**

Artificial intelligence (AI) offers potential solutions to address the challenges faced by a strained mental health care system, such as increasing demand for care, staff shortages, and pressured accessibility. While developing AI-based tools for clinical practice is technically feasible and has the potential to produce real-world impact, only a few are actually implemented into clinical practice. Implementation starts at the algorithm development phase, as this phase bridges theoretical innovation and practical application. The design and the way the AI tool is developed may either facilitate or hinder later implementation and use.

**Objective:**

This study aims to examine the development process of a suicide risk prediction algorithm using real-world electronic health record (EHR) data through a qualitative case study approach for clinical use in mental health care. It explores which challenges the development team encountered in creating the algorithm and how they addressed these challenges. This study identifies key considerations for the integration of technical and clinical perspectives in algorithms, facilitating the evolution of mental health organizations toward data-driven practice. The studied algorithm remains exploratory and has not yet been implemented in clinical practice.

**Methods:**

An exploratory, multimethod qualitative case study was conducted, using a hybrid approach with both inductive and deductive analysis. Data were collected through desk research, reflective team meetings, and iterative feedback sessions with the development team. Thematic analysis was used to identify development challenges and the team’s responses. Based on these findings, key considerations for future algorithm development were derived.

**Results:**

Key challenges included defining, operationalizing, and measuring suicide incidents within EHRs due to issues such as missing data, underreporting, and differences between data sources. Predicting factors were identified by consulting clinical experts; however, psychosocial variables had to be constructed as they could not directly be extracted from EHR data. Risk of bias occurred when traditional suicide prevention questionnaires, unequally distributed across patients, were used as input. Analyzing unstructured data by natural language processing was challenging due to data noise, but ultimately enabled successful sentiment analysis, which provided dynamic, clinically relevant information for the algorithm. A complex model enhanced predictive accuracy but posed challenges regarding understandability, which was highly valued by clinicians.

**Conclusions:**

To advance mental health care as a data-driven field, several critical considerations must be addressed: ensuring robust data governance and quality, fostering cultural shifts in data documentation practices, establishing mechanisms for continuous monitoring of AI tool usage, mitigating risks of bias, balancing predictive performance with explainability, and maintaining a clinician “in-the-loop” approach. Future research should prioritize sociotechnical aspects related to the development, implementation, and daily use of AI in mental health care practice.

## Introduction

Advances in artificial intelligence (AI; see Textbox 1 for a glossary of key terms) may provide promising opportunities for many actual and future challenges that mental health care faces [1], such as increasing demand for care and staff shortages that affect the quality of care and accessibility of mental health care. AI may enhance multiple domains, such as triage and screening, diagnostics, clinical decision-making, care delivery, chronic care management, self-care, and prevention of (psychiatric) symptoms [2].

Textbox 1. **Glossary.**

Artificial intelligence refers to a computerized system (hardware or software) that is able to perform tasks or reasoning processes that we usually associate with intelligence in a human being [3,4].

Machine learning is part of artificial intelligence. It is able to build algorithms that can detect patterns in large training datasets to explain variation in data or predict outcomes (predictive algorithms) [3,5-7].

Predictive analytics aims to build models which allow for individual (ie, single subject) predictions, thereby moving from the description of patients (hindsight) and the investigation of statistical group differences or associations (insight) toward models capable of predicting current or future characteristics for individual patients (foresight), thus allowing for a direct assessment of a model’s clinical use [8].

Natural language processing refers to ML techniques capable of parsing unstructured texts, such as free written texts or spoken language [9]. Natural language processing enables the use of unstructured data that is otherwise largely inaccessible, such as the dynamic fluctuations of symptoms (eg, by analyzing clinical notes) and can identify social and behavioral factors, which are not commonly registered in EHR [10].

Predictive algorithms are a promising subfield of AI, in which machine learning (ML) models generate predictions on an individual patient level to support decision-making in the diagnosis, prognosis, and treatment of patients [5,8,11]. Predictive algorithms have been shown to be more successful than traditional statistical models, as they provide a new way to gain knowledge from a data set. For example, in the field of predicting suicide risk at the individual level, predictive algorithms can yield more accurate predictions [12-15] than traditional statistical models, which predict only slightly better than chance and whose predictive ability has not improved over time, even after 50 years of research [16]. Not all ML models for suicide risk are adequate, although, as performance can vary depending on various factors, such as data quality, nature of the features used as input, preprocessing steps, and the used techniques [12]. In other mental health care domains, ML models are also able to perform better than traditional models, such as the prediction of psychosis, treatment response [17], or opioid use disorder [18].

Algorithms designed for clinical care often use patient data collected during regular clinical care in the organization’s electronic health record (EHR). Combining the expanding possibilities of AI with EHR data fuels the transformation into a data-driven mental health care, as the EHR contains rich, real-world, and real-time data. Reusing EHR data to develop algorithms that support clinical practice on an everyday basis opens many possibilities. However, it may be challenging, as these data are usually noisy, heterogeneous, dynamic, and contain both structured and unstructured data (eg, text) and require careful preprocessing [19]. Developing [20] and implementing AI-based algorithms using real-world EHR data has been proven to be feasible and can have a significant clinical impact [21-23]. However, the studies of algorithms that are actually implemented and used in mental health clinical practice are still scarce [24]. Also, there is a gap between the creation of scientifically sound algorithms and their meaningful application in real-world settings, a challenge often referred to as the "AI chasm" [25].

For mental health care to benefit from AI opportunities and the wealth of EHR data, developing algorithms based on real-world EHR patient data intended for clinical use is a crucial first step toward successful implementation and use. The development phase bridges theoretical innovation and practical application. Therefore, the development phase is not only a preparatory step but an integral part of implementation, as the design and development of the AI tool may either facilitate or hinder later implementation and use (eg, through alignment with organizational workflows and engendering trust) [26].

To enable the successful development, implementation, and actual use of AI and ML in mental health clinical practice, implementation science provides a valuable framework [3], as it studies the contextual factors that enable the process of embedding and integrating new practices into actual care [27,28]. Also, it fosters greater attention for the sociotechnical aspects of technology, focusing not only on the development of technological improvements but also the social changes required for their successful implementation and use [17,29-33]. Multiple barriers and facilitators have been identified in the literature, highlighting aspects of AI technology itself (eg, accuracy, transparency, and generalizability); organizational factors (eg, integration with workflows); governance, ethical, and legal issues; perception of staff and patients; or the implementation process (eg, engagement, user-centered design) [34,35]. However, these barriers and facilitators have, for a large part, been identified not primarily on implementation data but rather based on hypothetical implementation, white papers, and thought articles [34,35]. Therefore, more empirical studies are required to fill this knowledge gap [6,17].

To better understand how this development phase unfolds within the specific context of mental health care, we conducted a case study. This study explores the challenges encountered by a development team in creating a suicide risk prediction algorithm using real-world EHR data for clinical application in close collaboration with clinicians, as well as the team’s responses to these issues. Ultimately, we aim to identify key considerations relevant to bridging technical and clinical needs in algorithm development, offering insights for mental health professionals and organizations seeking to move toward data-driven care.

## Methods

### Research Design

This study is a detailed case study aimed to explore the challenges involved in the development of a suicide risk prediction algorithm using real-world EHR data in mental health care. Using an exploratory, multimethod approach with both inductive and deductive analysis suited to this emerging field, as the study seeks to identify key development considerations applicable to broader contexts [36].

### Case Description

#### Setting and Background

Suicide prevention in the Netherlands is organized through a national policy agenda focused on public awareness, early detection, and professional care. The national helpline 113 Suicide Prevention is available 24/7 by phone or chat. This organization also provides public campaigns and training for professionals and citizens. The National Suicide Prevention Agenda, developed by the Ministry of Health, Welfare and Sport together with 113 and other partners, offers a framework for collaboration between health care, education, municipalities, and the media. Locally, Municipal Health Services, youth health care, schools, and social services work on prevention and early risk identification. In mental health care, suicide prevention is part of standard practice, including staff training and procedures following a suicide incident. The Health and Youth Care Inspectorate monitors suicide prevention and reporting, while knowledge institutes, such as Trimbos, support professionals through guidelines and research.

GGz Eindhoven and the Kempen (Geestelijke Gezondheidszorg Eindhoven en de Kempen [GGzE]) is a mental health institution in southern Netherlands, providing specialized care to around 17,000 patients annually. At GGzE, a protocol is in place in accordance with the national multidisciplinary guideline [37]. All health care professionals are required to complete suicide prevention training courses. Suicide risk assessments are conducted at the start of each treatment trajectory and at other critical moments, for example, during a crisis; at the beginning, end, and after hospitalization; or prior to the extension of patient privileges during admission. A standardized suicide assessment tool, the Suicide Prevention Questionnaire (SPQ), is embedded within the EHR and should be administered at these key points, at least once or twice per year.

To enhance suicide prevention, GGzE explored the feasibility of developing a predictive algorithm using EHR data to support clinicians in identifying suicide risk in individual patients. Key objectives included establishing a reliable measure of suicide incidents, identifying predictive factors, and creating an algorithm with an accuracy target of 70%‐90% [38]. The development team, consisting of 2 data scientists with support from data science, governance, law, and clinical suicide experts (multiple psychologists and psychiatrists), operated under a structured project organization with a steering committee and project group. Both the steering committee and the project group comprised health care professionals as well as staff members with legal, organizational, and other forms of specialized expertise.

The project was embedded in the organization-wide Suicide Prevention Program, which provided consultation and training, developed organizational guidelines and protocols, organized evaluation of suicide incidents and subsequent quality enhancement of clinical practice, ensured evidence-based treatment options regarding suicidality, and developed a reflective learning culture regarding suicidality within the organization. The organization was part of the Suicide Prevention Action Network (Supranet GGZ), in which mental health institutions work together to make the quality of care for suicidal patients transparent, to learn what can be improved, and to improve care where necessary. Within this network, data on suicide incidents were structurally collected and shared, including core indicators on suicide risk.

#### The Suicide Prediction Algorithm: Description and Performance

The development team designed the algorithm for hospitalized patients with severe mental illness, using the Cross-Industry Standard Process for Data Mining (CRISP-DM) framework [39], a standard method in data mining and ML, to predict suicide risk within 1 month. Data for the algorithm was taken from the EHR and included data from 5044 patients (December 31, 2015, to August 31, 2021) included demographics (eg, age and sex), administrative details (eg, financing type), treatment history (eg, hospitalization count), psychiatric factors (eg, primary diagnosis), current treatment details (eg, duration and intensity), and social factors (eg, social contacts). Consultations with clinicians, interviews, and a literature search resulted in a list of possible predictors of suicide incidents that were derived from the EHR.

The team applied natural language processing (NLP) to clinicians’ daily reports, identifying unreported suicide incidents and capturing patient sentiment as an additional predictor, using topic modeling (BERTopic) [40]. Ensemble learning (extreme gradient boosting) [41] strengthened the model. The development team deemed the algorithm successful based on Hicks et al criteria [38]. The team acknowledged that the performance indicator outcomes were exploratory, as the algorithm would be retrained prior to implementation in a pilot phase and subsequently retrained annually in accordance with quality standards.

The predictive algorithm for suicide incidents will be gradually tested in a small-scale pilot project conducted in close collaboration with health care professionals. The algorithm will be implemented solely as an add-on to existing suicide prevention policies. Health care professionals will remain fully responsible for clinical assessment and decision-making. The pilot project will be evaluated at multiple stages throughout its implementation.

### Sampling and Participants

The study’s participants included 2 core data science professionals who led the development of the suicide risk prediction algorithm, supported by internal and external experts. The team worked closely with clinicians (multiple psychologists, psychiatrists, and the Medical Director’s office) and experts in data science, law, and data governance, as well as with external data science collaborators from other mental health care institutions. The participants were selected by expert sampling, in which participants are included who have demonstrable experience and expertise regarding the study’s aim and objective [42,43]. These participants offered crucial insights into the challenges and considerations of algorithm development. The team worked closely with clinicians (eg, psychologists, psychiatrists, and the medical director) and experts in data science, law, and data governance, as well as with external data science collaborators from other mental health care institutions.

### Data Collection and Data Analysis

Data for our study on the development process were collected through desk research, reflective meetings, and iterative feedback sessions with the development team. The study proceeded in three phases: (1) desk research and meetings with the development team to draw up a detailed process description, (2) identification of challenges and responses by thematic analysis, and (3) formulation of key considerations.

The detailed process description formed the basis for thematic analysis, which was conducted following the Braun and Clarke approach [44]. An exploratory, multimethod design combining inductive and deductive analysis was used to identify challenges during the algorithm development process. Deductive analysis was guided by the innovation domain of the Consolidated Framework for Implementation Research (CFIR) [28,45] and concepts from AI-related literature.

Data on process steps, challenges, and responses were analyzed and organized in a chart by 1 researcher (LH). Data was discussed by all 4 researchers to derive the main challenges and describe the team’s responses. Communicative validation was achieved through discussions with the development team. Triangulation across data sources strengthened the credibility and transferability of findings. Data collection and analysis were iterative and integrated throughout all phases.

### Ethical Considerations

Ethical approval was obtained from the Ethical Review Board of Tilburg University (reference TSB_RP1038). Participants received no financial compensation and provided written informed consent prior to participation. All procedures adhered to principles of privacy and confidentiality: no identifying information was collected or published, and any nonessential identifying details were omitted. No images or supplementary materials contain identifiable participant features.

## Results

### Overview

The encountered challenges and the team’s responses are described below, related to the construction of the suicide incidents (dependent variable) and possible predictors (independent variables), and the building of the model. At the start, the concept of the suicide incidents and the predictors had to be defined, operationalized, and measured. Several challenges were encountered during this phase: data was hard to find, data sources yielded different results, and underreporting was suspected. Regarding predicting factors, psychosocial predictors were not available and had to be estimated. To calculate the algorithm, the team used complex models that improved predictive performance but reduced understandability for clinicians.

Regular consultations between the project team and clinical experts during the development process were important to gain an accurate and profound understanding of the phenomena of suicide incidents and their predictive factors.

The Results section will focus on the development team’s challenges and their responses to them. In the Discussion section, the results will be contextualized within the literature to formulate key considerations for algorithm development in mental health care.

### Defining, Operationalizing, and Measuring Suicide Incidents

#### Different Data Sources Provide Different Outcomes

The data science team collaborated with health care professionals to define suicide incidents as the dependent variable. Within the organization, a suicide incident was defined as either a fatal suicide or a nonfatal attempt with a certain intention to die. This also covered self-harming or risky behavior that involved a risk of death or failure to avoid such risk. Severe suicide incidents were those that were fatal or caused, or could cause, severe or permanent physical injury [source: GGzE Protocol Reporting suicides and suicide attempts; consulted 2025-09-19]. Clinicians had to distinguish these from self-harm driven by other motives, such as reducing emotional pain.

Organizational protocols prescribed the registration of every suicide incident in a digital registration system. The Medical Director’s office kept its own registration. The 2 data sources within the organization yielded different outputs on the total number of suicide incidents:

*In the Client Details source there is a variable called Suicide Attempt. In addition, a variable called Suicide Incident has been created based on the incident data. […] there are more numbers […] for Suicide Incident than for Suicide Attempt.”* [Report of the development team and external partners on characteristics of the suicide incident risk algorithm, p. 10].

The medical director’s office clarified to the data science team that they reviewed the registered suicide incidents in the reporting system and recorded only those suicide incidents classified as severe. The team chose to include all suicide incidents, providing more data to train the algorithm and enabling the prediction of both severe and less severe cases. Clinical experts reflected on the overall numbers and considered them to be low, suggesting underregistration, particularly of less severe cases, possibly due to noncompliance with protocols or sensitivities around suicide. Moreover, ambiguity about the intention behind the behavior in less severe cases may have played a role. For example, behavior without serious injury or risk of injury that primarily served a communicative function.

#### Using Unstructured Data to Improve Detection of Suicide Incidents Is Challenging

To detect underreported suicide incidents, the development team added text mining using NLP of clinicians’ unstructured daily reports. However, the method produced significant data noise, identifying many reports related to suicidality but failing to pinpoint actual suicide incidents. Text analysis was hindered by clinicians’ writing habits, such as negation terms (“not feeling suicidal”), the use of synonyms (eg, “jumping off a bridge”), and the use of standard templates (eg, “has the patient expressed suicidality today?”).

Manual labeling of cases was tried, but appeared to be very time-consuming and unfeasible as a permanent solution, especially as the algorithm had to be retrained every 6 months due to quality requirements:

We still need to be 100% sure that there is/isn’t a suicide incident in the daily report data. Possible solution: Read the data yourself and assess whether there is/isn’t a suicide incident (label data). Disadvantages: […] Takes a lot of time (for a small dataset it took 3 days)

The development team responded to these challenges by making efforts to enhance data quality. The organization was in the process of transitioning to another provider of an EHR system. The team recommended including a check mark for suicide incidents [source: Presentation Suicide Predictive Model_2^nd^ iteration, p. 36] within this new system and advocated this solution in the new EHR project team, underlining the importance of these interventions to transform into a data-driven health care organization. Also, a new executive board took the lead of the organization and, consequently, a new multiyear strategy was developed, in which “working data-driven to enhance decision support” was one of the main ambitions [source: GGzE, multiyear strategy 2024‐2028; consulted 2025-09-19]. This prompted management to take a more active role in ensuring data quality, such as completing suicide-related information. Also, the organization implemented an alternative instrument for the SPQ. This way, the organization aimed to improve data quality. The algorithm would be retrained when the new EHR system was implemented.

However, the team still had to deal with the fact that possible underregistration of suicide incidents, especially less severe cases, was an issue. From a data science perspective, they could interpret this as missing data, which threatened data quality. From a clinical perspective, the possibility that mild incidents were not always reported was a reflection of the ambiguity of the interpretation of a suicide incident: did the patient actually aim to end their life, or should the behavior be interpreted more as self-harming behavior with the intent to decrease pain or to communicate distress?

### Defining, Operationalizing, and Measuring Potential Predicting Factors

The development team compiled a list of potential predicting factors by performing a literature review, reviewing factors from well-known suicide risk assessment methods, and interviewing clinicians to leverage their expertise and to involve and engage clinicians in the development process. The list of predictors then had to be defined, operationalized, and measured. Reflective meetings with the development team revealed several challenges they faced, which are described below.

#### The EHR Lacks Psychosocial Factors: Construction of Predictors is Necessary

Some predicting factors, like previous suicide attempts or substance dependence, could be directly derived from the EHR. Other predicting factors were psychosocial and could not be directly obtained from the EHR, thus requiring the construction of an estimator.

For example, information on the number of contacts of a patient, as registered in the EHR, along with a selection of characterization of these relationships (eg, spouse, sibling, child, and neighbor) was used to construct the factor “social network.” Also, the factor “quality of the therapeutic relationship” was operationalized as the number of primary [1] practitioners during hospitalization. Although it was recognized that these operationalizations did not necessarily reflect the actual factors, in the absence of a better alternative, it was decided to operationalize and measure these concepts in this way.

#### Data Availability Differs Between Subgroups: Risk of Bias

Clinicians considered the SPQ, available in the EHR, the most promising source of predictors. Although protocols required annual administration for all patients, the data science team found a recent SPQ in only 21.95% of cases. Clinicians confirmed that SPQ variables were highly explanatory but acknowledged their limited availability. They suspected that SPQs were often completed only when suicide risk was already suspected, creating bias and overrepresenting high-risk patients. To include all patients, the data science team had to derive estimators from other EHR data. Additional disparities emerged: SPQs were missing more often for youth (0‐17 y) than for older patients, despite comparable incident rates. These findings were shared with the Child & Youth Department to improve data quality. Ethnicity was also explored as a predictor but was excluded due to frequent missing values.

Overall, the results exposed a gap between organizational protocols (eg, annual SPQ administration, registration of ethnicity) and actual practice. They also revealed different perspectives: clinicians valued SPQ data but were less familiar with data science principles, such as representativeness and external validity, while data scientists emphasized these risks. Close collaboration was deemed essential to identify predictors and minimize bias.

To address these challenges, the development team worked with the Suicide Prevention Program and a national suicide prevention organization to design a digital toolkit as a replacement for the SPQ. This decision support tool aimed to make risk assessment more user-friendly, link assessments to targeted treatment advice based on the national guideline, and reduce clinician workload through robotic process automation that transferred data directly to the EHR.

#### Sentiment Analysis to Construct a Time- and Context-Sensitive Predictor

As the literature review and interviews with clinicians emphasized the role of time- and context-related factors in suicide risk (eg, relationship breakup and intoxication), the development team explored ways to integrate these into the model. Since collecting daily data directly from patients was impractical due to severe psychiatric conditions and low cooperation, daily reports by clinicians about patients were identified as the best alternative. These allowed real-time data to strengthen predictive accuracy.

Although detecting suicide incidents in daily reports was challenging, sentiment analysis proved feasible. Patients with suicide incidents showed lower sentiment in the daily notes by clinicians, especially in the most recent entries, compared to those without incidents (internal document: PowerPoint Suicide Predictive Model_2nd iteration*,* p. 29).

When the development team presented the results to the medical director’s office, new applications were discussed. The algorithm was initially designed as an add-on to existing protocols, such as administering an SPQ annually. Given the time-intensive nature of the SPQ, 1 proposal was to let the algorithm preselect high-risk patients for SPQ administration. This would save time by limiting assessments to flagged patients.

Clinical experts, including the medical director, rejected this option, noting that the algorithm was still in development and should not replace established tools. They did, however, acknowledge that SPQs were not consistently administered in practice. As an alternative, they proposed using sentiment analysis as an alert system: if emotional decline was detected and no recent SPQ was available, clinicians would be prompted to administer one. This approach was accepted, as it integrated the algorithm while reinforcing compliance with existing suicide prevention protocols.

### Building the Algorithm: High-Performance Model Appears Hard to Understand by Clinicians

After identifying dependent and independent variables, the development team built the algorithm using ensemble learning, which combines weaker models into a stronger one. This approach delivered high predictive power, meeting the project’s feasibility goal, but the model was too complex to interpret directly.

The data science team shared the results with clinicians and the steering group, including the model’s performance metrics (eg, accuracy, precision, and recall), acknowledging that the results were exploratory as the model would later be retrained. Clinicians appreciated the potential value of the algorithm but emphasized that understanding why suicide risk was elevated was essential for fostering trust, ensuring accountability, and supporting treatment planning. They highlighted that a well-performing algorithm alone would not be sufficient to generate meaningful benefits in clinical practice. The data science team, therefore, identified explainability as a core requirement. They aimed to develop case-level reports that not only flagged risk but also explained the reasons behind it: “Not only: alarm! But also: alarm, for this reason” [Reflective meeting, 2023-11-07].

To address this challenge, a team member engaged in additional training in explainable AI, noting the scarcity of knowledge within mental health care. Additionally, the data science team planned to organize several collaborative sessions with clinicians to cocreate an explainability dashboard that contextualizes the algorithm’s outputs. Also, collaborative sessions were planned to facilitate the integration of the algorithm into existing workflows and jointly reflect on the tasks, roles, and responsibilities of each stakeholder in its use.

## Discussion

### Principal Findings

This study provided a detailed case study of the development of a suicide risk prediction algorithm using real-world EHR data. Its unique contribution lies in demonstrating how algorithm development is carried out within the clinical practice of mental health care, where technical innovation must be aligned with clinical needs, professional values, and organizational structures. By describing the challenges of a development team and their responses to them, this study offers an in-depth description of the development of a predictive algorithm, in which the development process is seen as a first step in the implementation process.

This paper highlights the sociotechnical and organizational aspects of algorithm development, showing how collaboration, joint efforts to enhance data quality, and clinician engagement are essential prerequisites for algorithm development.

### Reflection on the Principal Results

#### Addressing Data Quality Challenges in the EHR

High data quality is essential for AI in mental health care, yet EHR data are often low in quality, incomplete, “soft,” and indirect [46,47]. Also, handling unstructured EHR data requires unique approaches [48,49].

Regarding data quality, EHR data quality in mental health is typically problematic. In the current case, suicide incident data were hard to construct and possibly underreported, although this could also be a reflection of real-world clinical assessment issues. Low data quality is noted in the literature, where suicide incidents are frequently underdocumented [50] due to clinicians’ workload [7,46] and departmental workflow inconsistencies [51]. Addressing this, data quality issues span all 5 dimensions identified by Weiskopf and Weng [52]: completeness, correctness, concordance, plausibility, and currency. Organizations are faced with challenges on data quality according to each of these dimensions.

Mental health data often rely on “soft” psychological constructs (eg, social support), which are harder to quantify than “hard” somatic measures (eg, heart rate) [47]. This raises risks for construct validity [53]. Moreover, much of the data reflects professionals’ perceptions rather than patients’ own, which may diverge significantly [54-57]. Future approaches using direct patient data (eg, real-time sampling and devices) could help improve accuracy [13].

Analysis of unstructured data, like staff notes, showed high noise, with frequent mentions of suicidality but few actual incidents, a challenge also noted in other mental health domains [13,58]. Variations in writing style and negations further complicate analysis [59-61]. Still, NLP offers potential to capture real-time risk fluctuations and enhance predictive accuracy [62-65].

While low data quality in mental health is a barrier for AI, algorithm development may also support quality improvement. In the current case, feedback on data inconsistencies spurred organizational motivation to enhance quality and adapt the EHR system.

#### Mitigating Risk of Bias to Ensure Performance for Subgroups

When algorithms are trained on data of low quality, risk of bias (RoB) occurs, in which the algorithm may not perform as well for each subgroup, and human biases are repeated and even amplified. When predictors are constructed from EHR data as data are not readily available, selection bias may occur. Algorithms require balanced representation across subgroups, though, to perform effectively. In this case, using SPQ items as predictors risked overrepresenting high-risk patients and weakening performance for others. While EHR data enables inclusion of all hospitalized patients, including minority groups often underrepresented in research [66], bias remains a major concern. As social factors are often key predictors in mental health care [15,67], social data is often missing due to true absence or nonrandom issues, such as patient refusal, confidentiality concerns, or language barriers, and excluding such cases may worsen bias [15,67]. Selection bias also threatens performance when samples do not reflect the broader population [10,68,69]. Bias is therefore considered an additional dimension of data quality, alongside the 5 identified by Weiskopf and Weng [52,70].

By building algorithms from reused EHR data, human biases in the data may be reinforced. The development team acknowledged this risk but considered the potential benefits sufficient to proceed, provided mitigating measures were applied. Since algorithms are trained on subjective EHR data, cognitive biases, such as stereotypes or in-group favoritism [71-73], may become embedded and systematically applied [66,68,69]. In practice, if an algorithm’s outcome aligns with a clinician’s initial diagnosis, confirmation bias may lead the clinician to ignore contradictory information. If the outcomes differ, precautionary bias might cause them to trust the algorithm over their own judgment [73]. Automation bias could also emerge, where clinicians rely more on the tool than on other information, potentially diminishing their skills [7]. The question remains what mitigating measures are adequate to prevent systematically disadvantaging subgroups. At the same time, algorithms may also reduce bias by integrating fragmented information across professionals and systematically processing both subjective and objective data [74]. Determining under which conditions algorithms amplify or mitigate bias remains an open question for future research.

#### Actionability, Trust, Accountability, and Communication in Complex Models

Predictive power and understandability have to be balanced in algorithm development. In our study, data scientists prioritized predictive performance, applying a complex model to enhance accuracy, while clinicians emphasized the need for understandability. This reflects the broader trade-off between complex “black box” models, which yield higher predictive power but limited explainability, and simpler “glass box” models, which are more interpretable but often less accurate [75-77]. Initially, the team focused on feasibility, whether an algorithm with sufficient predictive performance could be built from organizational data, rather than on clinical applicability. Clinician feedback prompted the inclusion of explainability as a key objective, as it enhances the actionability of the outcomes, that is, whether clinicians can use the information to identify actions to change the outcome predicted by the algorithm [78]. In suicide prevention, detecting elevated risk is insufficient; clinicians must also understand which factors contributed most, which are modifiable, and which interventions are likely to reduce risk [79,80]. Explainability is crucial for building trust [81-83], and providing an appropriate level of transparency (not too little and not too much) is necessary in designing for trust [84]. Clinicians should be well informed about the actual capabilities of the algorithm, as responsible use of AI systems also depends on the level of correspondence between a user’s trust and the technology’s capabilities, known as calibration [83]. Explainability may also enhance patient communication and support shared decision-making [76,79,85]. Also, mental health institutions are accountable for care quality, effectiveness, and safety. In our study, clinicians feared that using complex, nonexplainable models would undermine accountability, as evidence-based protocols are easier to justify than black-box algorithms. Given the high-stakes nature of suicide care, caution is warranted when applying complex models [80].

Whether performance or explainability should be prioritized in mental health care remains unresolved, and future research should examine context-specific trade-offs as well as post hoc strategies to enhance explainability [77,86]. Future research should explore what information should be communicated to clinicians and how it should be presented. Moreover, fostering mutual trust and collaboration between clinicians and data scientists throughout the algorithm development process is essential. Technical performance alone does not guarantee clinical usefulness. Instead, cocreation of both the algorithm and its user interface, combined with joint efforts to integrate it into everyday practice, is crucial for enabling clinicians to trust and meaningfully use the algorithm’s outcomes.

Below, we present Table 1 summarizing the challenges encountered during the development phase, the corresponding responses by the team and organization, and the broader considerations derived from these experiences.

**Table 1.** Challenges, responses, and considerations during algorithm development in mental health care.

The development phase	Challenges	Responses	Considerations
Defining, operationalizing, and measuring suicide incidents	Inconsistent detection of suicide incidents across data sources.	Enhanced suicide incident data by integrating clinician insights. Broadened incident inclusion. Tested NLP^a^ approaches. Improved EHR^b^ registration.	Ensure sufficient data availability for the predicted outcome. Consult clinicians to improve data interpretation. Consider clinical explanations for data quality issues. Provide user-friendly EHR options for registering suicide incidents.
Defining, operationalizing, and measuring potential predicting factors	Limited and uneven availability of psychosocial and contextual predictors constrains robust and unbiased model development.	Developed high-availability estimators. Addressed data gaps with clinicians. Implemented a guideline-aligned assessment tool to simultaneously enhance data registration and support clinical decision-making. Developed a sentiment analysis for contextual risk signals.	Ensure predictor validity by prioritizing high-availability EHR data. Address subgroup gaps through data governance. Supplement psychosocial information via clinicians or patients. Develop user-friendly assessment tools that integrate required data registration with clinical decision-making support.
Building the algorithm	Complex models improve prediction but are difficult for clinicians to interpret.	Established explainability as a core requirement. Planned cocreation of an explainability dashboard with clinicians before the pilot.	Balance predictive performance and explainability through close collaboration between data scientists and clinicians.
Toward an implementation pilot	Effective use requires full integration into clinical workflows and sufficient clinician experience. Algorithm use may introduce cognitive and decision biases.	Planned joint data scientist–clinician sessions to integrate the algorithm into workflows and clarify roles. Positioned the algorithm as an add-on to suicide prevention care, with clinicians’ judgment remaining central.	Organize data scientist–clinician sessions to support workflow integration and clarify roles. Clearly communicate the algorithm’s role and its support function for clinical decision-making. Calibrate clinicians’ expectations to the algorithm’s actual capabilities and limitations.

a NLP: natural language processing.

b EHR: electronic health record.

### Limitations

This study provided a detailed case study of the development of a suicide risk prediction algorithm using real-world EHR data, highlighting the tension between technical requirements and clinical needs. Using a qualitative, real-world approach, the study captured contextualized knowledge from the development team, who collaborated closely with clinicians. While generalizability is limited by the single-case design, triangulation and detailed process descriptions support transferability. A key limitation is that clinicians’ perspectives were obtained indirectly via the development team rather than through direct engagement, although this was partly mitigated by desk research and targeted questions to include broader stakeholder views.

### Conclusions and Future Research

#### Conclusions

Based on the findings of our case study, we highlight the following 5 main recommendations for mental health organizations that aim to evolve into data-driven practice.

1. Data governance and quality: high-performing algorithms require robust data governance and timely, deliberate data preparation to ensure data quality.
2. Cultural change in data entry: a cultural shift is needed to regard and manage data as a strategic asset for algorithm development.
3. Bias awareness and monitoring: organizations must remain sensitive to potential bias in both data and use, requiring careful data handling and regular evaluation of real-world application.
4. Balancing performance and transparency: predictive performance using complex models must be balanced with clinicians’ needs for transparency and explainability to ensure actionability, trust, communication, and accountability.
5. Clinician-in-the-loop: it is essential to define the knowledge, skills, and workflows that enable clinicians to use algorithms responsibly while maintaining final clinical judgment.

For mental health organizations aspiring to evolve toward data-driven care, early preparation is essential. First, building predictive algorithms requires sufficient and well-structured data, which demands timely and deliberate data collection. Reliance on existing organizational protocols alone is insufficient; explicit attention must be given to how relevant variables are defined, recorded, and embedded in the EHR system. A cultural transformation is also critical. Second, data should not be viewed merely as an administrative or accountability tool but as a strategic resource for predictive modeling. However, this is not always aligned with clinical practice and may require a change in working processes for clinicians. This transition requires awareness and commitment from clinicians, whose engagement from the earliest stages of algorithm development is crucial. Collaboration between data scientists and clinicians must begin in an early stage of the development process: data scientists contribute technical and data-analytical expertise, while clinicians bring contextual understanding, interpret data inconsistencies, identify meaningful predictors, and help foster a data-driven culture. Organizations must also recognize potential biases within their data and proactively assess how information from subgroups is collected and represented. Third, ongoing attention to bias is crucial, as it may stem not only from data or model design but also from real-world use. Regular evaluation of both performance and application is essential to ensure quality and responsibility in practice. Decisions regarding model design should balance predictive accuracy with interpretability. Fourth, explainable AI techniques may help translate complex model outputs into clinically meaningful insights, allowing clinicians to understand and act upon algorithmic predictions. Clinicians require not only accurate models but also transparency to understand the reasons for the outcome to ensure actionability, trust, and clinician-patient communication. Institutions should further consider how accountability for algorithm use is shared between individual practitioners and the organization. Finally, advancing toward data-driven mental health care requires investment in clinicians’ education and professional development regarding AI. Defining the knowledge, skills, and workflows that enable responsible algorithm use, while maintaining clinical judgment as the ultimate decision point, will be key to realizing the added value of predictive technologies in practice.

#### Future Research

Future research should further explore how robust data governance structures can be established to ensure data quality and readiness for algorithm development. This includes examining how AI techniques may contribute to improving data quality through automation, standardization, and enhanced data validation. Moreover, studies should investigate how cultural change can be fostered to promote viewing data as a strategic resource rather than an administrative by-product, and how such shifts can be maintained throughout the organization.

Attention to bias should extend beyond data collection and model design to the actual use of algorithms. Future studies are needed to determine under which conditions algorithms amplify or mitigate bias, and how systematic monitoring of real-world application can safeguard fairness and responsible use.

The ongoing tension between predictive performance and explainability also warrants investigation. Research should identify context-specific trade-offs between complex, high-performing models and simpler, more transparent approaches and explore how explainability dashboards can enhance actionability, trust, accountability, and clinician–patient communication.

Finally, future research should examine what knowledge, skills, and workflows clinicians need to engage responsibly with predictive algorithms while maintaining final clinical judgment. Integrating sociotechnical considerations throughout algorithm development will be key to ensuring that AI tools are both effective and ethically grounded in clinical practice.

While much focus has been placed on the technical aspects of ML in mental health care, sociotechnical factors related to successful algorithm development, implementation, and use have been largely overlooked [6,13,15,17,24,25,30,33,87]. More research is needed to explore these issues in real-world settings to ensure responsible and sustainable innovation.

## Abbreviations

AI: artificial intelligence
CFIR: Consolidated Framework for Implementation Research
CRISP-DM: Cross-Industry Standard Process for Data Mining
EHR: electronic health record
GGzE: Geestelijke Gezondheidszorg Eindhoven en de Kempen
ML: machine learning
NLP: natural language processing
RoB: risk of bias
SPQ: Suicide Prevention Questionnaire
